# Ethnic discrimination, asking for fair treatment, and poor self-rated health: a gender stratified analysis of 13,443 Korean Chinese waged workers in South Korea

**DOI:** 10.1186/s12939-024-02160-0

**Published:** 2024-04-25

**Authors:** Hayoung Lee, Ji-Hwan Kim, Garin Lee, Hyelin Lee, Mita Huq, Delanjathan Devakumar, Seung-Sup Kim

**Affiliations:** 1 Department of Environmental Health Sciences, Graduate School of Public Health, Seoul National University, 1 Gwanak-ro, Daehak-dong, Gwanak-gu, 08826 Seoul, Republic of Korea; 2 Institute of Health and Environment, Seoul National University, Seoul, Republic of Korea; 3 Institute for Global Health, University College London, London, UK

**Keywords:** Ethnic discrimination, Asking for fair treatment, Coping, Self-rated health, Gender difference, Gender analysis, Korean Chinese, Joseonjok, Choseonjok

## Abstract

**Background:**

In South Korea, Korean Chinese workers experience ethnic discrimination although they share physical similarities and ethnic heritage with native-born Koreans. This study aimed to examine whether perceived ethnic discrimination is associated with poor self-rated health and whether the association differs by gender among Korean Chinese waged workers in South Korea.

**Methods:**

We conducted a pooled cross-sectional analysis using data of 13,443 Korean Chinese waged workers from the Survey on Immigrants’ Living Conditions and Labor Force conducted in 2018, 2020, and 2022. Based on perceived ethnic discrimination, asking for fair treatment, and subsequent situational improvement, respondents were classified into the following four groups: “Not experienced,” “Experienced, not asked for fair treatment,” “Experienced, asked for fair treatment, not improved,” and “Experienced, asked for fair treatment, improved.” Poor self-rated health was assessed using a single question “How is your current overall health?” We applied logistic regression to examine the association between perceived ethnic discrimination and poor self-rated health, with gender-stratified analyses.

**Results:**

We found an association between ethnic discrimination and poor self-rated health among Korean Chinese waged workers. In the gender-stratified analysis, the “Experienced, not asked for fair treatment” group was more likely to report poor self-rated health compared to the “Not experienced” group, regardless of gender. However, gender differences were observed in the group stratified by situational improvements. For male workers, no statistically significant association was found in the “Experienced, asked for fair treatment, improved” group with poor self-rated health (odd ratios: 0.87, 95% confidence intervals: 0.30–2.53). Conversely, among female workers, a statistically significant association was observed (odd ratios: 2.63, 95% confidence intervals: 1.29–5.38).

**Conclusions:**

This study is the first to find an association between perceived ethnic discrimination and poor self-rated health, along with gender differences in the association between situational improvements after asking for fair treatment and poor self-rated health among Korean Chinese waged workers in South Korea.

**Supplementary Information:**

The online version contains supplementary material available at 10.1186/s12939-024-02160-0.

## Introduction

Racial/ethnic discrimination is widely recognized as a social determinant of health [1–3]. Several systematic reviews have demonstrated an association between self-reported racial/ethnic discrimination and adverse health outcomes among diverse minority groups [4–7]. These outcomes encompass both physical and mental health, including cardiovascular disease, self-esteem, obesity, psychological stress, and depression.

While racial/ethnic discrimination in public health research commonly focuses on differences in physical appearance (e.g., skin color) as a reason for discrimination, previous studies have pointed out that it can also occur between populations lacking such differences. The experience of Japanese Brazilians, who immigrated to Brazil in the early 1900s in search of jobs, becoming the highest population of ethnic Japanese outside of Japan [8], offers a telling example. In the late 1980s, the Japanese government invited Japanese Brazilians back to Japan to address labor shortages in unskilled jobs [9]. Nonetheless, despite their shared physical appearance and ethnic heritage with native Japanese, these returnees have encountered negative and discriminatory treatment from native-born Japanese [10]. They were often considered cultural outsiders and relegated to lower socioeconomic positions in Japan [11]. Another study also found that the experience of ethnic discrimination from native Japanese people was associated with an increased risk of poor self-rated health and psychological symptoms among Japanese Brazilians [8].

In South Korea, the experience of Korean Chinese people offers a parallel example. Since the late 19th century, a number of Koreans migrated to China, either to escape famine or to establish a Korean government in exile against Japanese colonial rule (1910–1945) [12]. Many have settled in the Yanbian Korean Autonomous Prefecture, where they opt to preserve their Korean cultural heritage and language [13]. Since the 1990s, the South Korean government initiated short-term work visa programs targeting foreign manual laborers to address their labor shortage in low-skilled sectors [14]. Korean Chinese individuals, who largely share physical features and ethnic heritage with native Koreans and predominantly speak fluent Korean, emerged as the ‘preferred’ candidates for these programs. This led to a significant rise in return migration, with many Korean Chinese coming back to their ancestral land.

Korean Chinese returnees, initially expected to share cultural similarities with local Koreans due to shared descent and phenotype, eventually were perceived as ‘foreigners’ in South Korea [14]. They were legally barred from applying for the long-term stable visa programs [14] and were considered socially and culturally distinct from native-born Koreans [12]. Their extended separation from their ancestral homeland resulted in cultural differentiation from Korea [15], and their disadvantaged socioeconomic status often led to their cultural identity being viewed as inferior to native-born Koreans [12]. They were easily identifiable by their distinctive Korean accent and were often stereotyped as potential criminal suspects, undocumented foreign workers, or compatriots from impoverished countries [16, 17]. Furthermore, the neighborhoods where many of them reside are labeled as dangerous and ghettoized.

As of May 2022, Korean Chinese accounted for 36.8% of the 1,301,900 total foreigners aged 15 or older residing in South Korea, making them the largest migrant group [18]. Until the early 2000s, the majority of Korean Chinese return migrant workers held short-term work visas, such as the Visit and Employment Program (VEP) [19]. These visas permitted them to engage in manual labor, primarily within specific industries such as construction, manufacturing, and services. Since then, there has been a shift in South Korean immigration policy which has allowed these workers to obtain overseas Korean visas and attain permanent residency status upon meeting specific conditions, including a designated period of employment in sectors facing persistent labor shortages. Despite these policy changes providing prolonged employment opportunities and increased occupational freedom, the majority of Korean Chinese workers in South Korea still find themselves confined to low-wage and non-professional occupations that are often overlooked by native-born Koreans. Consequently, this occupational segregation places them in marginalized socioeconomic positions and alienates them from the majority of Koreans.

While several studies have focused on the harmful health effects of perceived racial/ethnic discrimination in South Korea [20–24], limited research has been conducted among Korean Chinese individuals in the country. To our knowledge, we found one study that identified a higher prevalence of depressive symptoms among Korean Chinese in South Korea who reported experiencing discrimination [25]. However, this study did not encompass the victims’ response to the discrimination, nor did it conduct a gender-stratified analysis.

Therefore, to fill this knowledge gap, the present study sought to answer the following questions: (i) Is there an association between perceived ethnic discrimination and poor self-rated health among Korean Chinese waged workers in South Korea? (ii) How does the association between perceived ethnic discrimination and poor self-rated health differ according to different responses to discrimination (whether or not asking for fair treatment) and subsequent situational improvements (whether or not the situation was improved after the response)? Does this association vary by gender?

## Methods

### Data and study population

This study analyzed data from the Survey on Immigrants’ Living Conditions and Labor Force (SILCLF) conducted by Statistics Korea and the Ministry of Justice in 2018, 2020, and 2022. SILCLF is a nationally representative survey conducted since 2017 to identify the actual conditions of foreign residents and naturalized citizens and to establish primary statistical data for immigration-related policies, such as residence management and social integration [26]. The target population of SILCLF is immigrants over the age of 15 who have stayed in South Korea for more than 91 days and those who have been naturalized within the last five years.

SILCLF is divided into two parts: a common set of questions for all immigrants and a specific set of questions for certain groups of immigrants depending on their visa program. The common survey covers three topics (basic socio-demographic information, employment, and residence) each year, with four additional topics (education, housing and living environment, income and expenditure, and education of children) in odd-numbered years and three additional topics (social participation, health and internet usage, and Korean language skills) in even-numbered years.

For this study, we aggregated three years of survey datasets from SILCIF to ensure the largest possible sample size for a statistically stable analysis of the association between discrimination, asking for fair treatment, situational improvement, and self-rated health after stratification by gender. The content of all variables intended for measurement remained consistent across each survey, and we ensured the matching of these variables in each survey dataset.

Among a total number of 64,077 nationally representative samples collected in the 2018, 2020, and 2022 surveys, we excluded those who were unemployed or economically inactive (*N* = 23,402), self-employed or unpaid family workers (*N* = 3,099), and workers on temporary leave (*N* = 571) to focus on waged workers in this analysis. Workers who had stayed in Korea for less than one year (*N* = 1,614) were excluded to measure experiences of ethnic discrimination within the past year. Finally, only Korean Chinese, including naturalized individuals, were included in the analysis. Naturalized individuals were included in the sample because they were naturalized recently (within the last five years), and the literature on returning Korean Chinese reported no significant differences in discrimination experiences after obtaining formal citizenship [27]. Consequently, a total of 13,443 Korean Chinese waged workers were included in our analysis. This research received IRB exemption from the Office of Human Research Administration at the Seoul National University (IRB No. E2310/003 − 002).

## Measurements

### Perceived ethnic discrimination and response to discrimination

We measured perceived ethnic discrimination by asking the following question: “Have you experienced any discrimination in the past year because of your status as a foreigner or as a naturalized citizen?” Workers could choose ‘Yes’ or ‘No’. Those who answered ‘Yes’ were identified as having experienced ethnic discrimination.

Asking for fair treatment was assessed by the follow-up question “Have you ever asked a person or an institution to stop discriminating against you?” Workers could answer ‘Yes’ or ‘No’. In addition, subsequent situational improvements were assessed by the follow-up question “Did the situation improve after you demanded the discrimination to stop?” Workers could answer either ‘Yes’ or ‘No’.

Workers who experienced discrimination were divided into three groups: a group that did not ask for fair treatment (“Experienced, not asked for fair treatment”), a group that asked for fair treatment but had no improvement (“Experienced, asked for fair treatment, not improved”), and a group that asked for fair treatment and had an improvement (“Experienced, asked for fair treatment, improved”).

### Self-rated health

Self-rated health was measured by the question “How is your current overall health?” Workers could answer on the following five-point Likert scale: 1 Very Good, 2 Somewhat Good, 3 Fair, 4 Somewhat Poor, and 5 Very Poor. The responses were classified into two groups: one with ‘good’ health ratings (responses 1 to 3) and the other with ‘poor’ ratings (responses 4 and 5).

### Covariates

Socio-demographic covariates included in the data analysis were gender, age, education, length of stay in Korea in years, residential area, monthly income, marital status, visa type, and Korean language ability. Gender was classified as male or female. Age was divided into five categories: 15–29, 30–39, 40–49, 50–59, and 60 years or more. Education was classified into four categories: elementary school graduate or less, middle school graduate or less, high school graduate or less, and college graduate or more. Length of stay in Korea was categorized into four groups: ≧1 & <3, ≧3 & <5, ≧5 & <10, and ≧10 years. Residential area was divided into two groups: metropolitan areas (including Seoul, Incheon, and Gyeonggi) and non-metropolitan areas. Monthly income was categorized into four groups: <1, ≧1 & <2, ≧2 & <3, and ≧3 million Korean won. Marital status was classified as currently married or currently not married. Visa type was classified into VEP, overseas Korean, permanent residency, marriage migrant, naturalized, etc. Korean language ability was assessed in four domains: speaking, listening, reading, and writing. Workers could answer on the following five-point Likert scale: 1 Very good, 2 Somewhat good, 3 Fair, 4 Somewhat poor, 5 Very poor. Scores from the four domains were summed, resulting summed scores ranged from 4 to 20. Workers were then divided into three groups: fluent (4–9), fair (10–15), and poor (16–20).

Four occupational covariates were included: employment type, company size, working hours per week, and occupation. Employment type was categorized into permanent, temporary, and day. Company size was divided into three groups: <5, 5–49, and ≧ 50 employees. Working hours per week were categorized into < 20, ≧20 & <30, ≧30 & <40, ≧40 & <50, ≧50 & <60, and ≧ 60 h. Occupations were categorized into white collar (manager, professionals, office workers), pink collar (service workers, sales workers), and blue collar (skilled agricultural and fishery workers, plant and machine operators and assemblers, and manual laborers).

Finally, the following survey years were included: 2018, 2020, and 2022.

### Data analysis

We estimated the prevalence of perceived ethnic discrimination and poor self-rated health by key covariates and compared the prevalence across different groups using a chi-square test. We used logistic regression to examine whether the perceived ethnic discrimination, asking for fair treatment, and subsequent situational improvements were associated with poor self-rated health among Korean Chinese waged workers. Further, we analyzed whether the association differed by gender.

All covariates were included as categorical variables in the analyses. The estimated results were presented as odd ratios (ORs) with 95% confidence intervals (CIs). All statistical analyses were conducted using STATA/SE version 17.0.

## Results

Table 1 shows the distribution of the study population, the prevalence of poor self-rated health, and perceived ethnic discrimination by key covariates. The overall prevalence of poor self-rated health was 8.6% (1,150 workers) and that of ethnic discrimination was 20.5% (2,762 workers).

**Table 1:** Distribution of study population, prevalence of poor self-rated health, and ethnic discrimination among Korean Chinese waged workers in South Korea by key covariates (*N* = 13,443)

	Distribution	Poor self-rated health		Ethnic discrimination	
	N (%)	N (%)	p-value^†^	N (%)	p-value^‡^
Overall	13,443 (100.0)	1150 (8.6)		2762 (20.5)	
**Gender**			< 0.001		0.610
Male	8316 (61.9)	493 (5.9)		1697 (20.4)	
Female	5127 (38.1)	657 (12.8)		1065 (20.8)	
**Age (years old)**			< 0.001		< 0.001
15–29	728 (5.4)	11 (1.5)		163 (22.4)	
30–39	2898 (21.6)	75 (2.6)		683 (23.6)	
40–49	3263 (24.3)	188 (5.8)		683 (20.9)	
50–59	4232 (31.5)	466 (11.0)		826 (19.5)	
60–	2322 (17.3)	410 (17.7)		407 (17.5)	
**Education**			< 0.001		< 0.001
Elementary school graduate or less	1394 (10.4)	214 (15.4)		262 (18.8)	
Middle school graduate or less	3333 (24.8)	350 (10.5)		643 (19.3)	
High school graduate or less	6507 (48.4)	499 (7.7)		1321 (20.3)	
College graduate or more	2209 (16.4)	87 (3.9)		536 (24.3)	
**Length of stay in Korea (years)**			< 0.001		0.164
≧1 & <3	734 (5.5)	32 (4.4)		138 (18.8)	
≧3 & <5	1407 (10.5)	87 (6.2)		316 (22.5)	
≧5 & <10	4615 (34.3)	332 (7.2)		957 (20.7)	
≧10	6687 (49.7)	699 (10.5)		1351 (20.2)	
**Residential area**			< 0.001		0.793
Metropolitan area	8591 (63.9)	802 (9.3)		1771 (20.6)	
Non-metropolitan area	4852 (36.1)	348 (7.2)		991 (20.4)	
**Monthly income (won)**			< 0.001		< 0.001
<1000 K	431 (3.2)	127 (29.5)		120 (27.8)	
≧1000 K & <2000 K	3050 (22.7)	429 (14.1)		703 (23.0)	
≧2000 K & <3000 K	6571 (48.9)	470 (7.2)		1303 (19.8)	
≧3000 K	3391 (25.2)	124 (3.7)		636 (18.8)	
**Marital status**			0.362		0.507
Currently married	9818 (73.0)	853 (8.7)		2031 (20.7)	
Currently not married	3625 (27.0)	297 (8.2)		731 (20.2)	
**Visa type**			0.297		0.135
Visit and employment program	2806 (20.9)	216 (7.7)		578 (20.6)	
Overseas Korean	5457 (40.6)	470 (8.6)		1117 (20.5)	
Permanent residency	1710 (12.7)	141 (8.2)		383 (22.4)	
Marriage migrant	303 (2.3)	29 (9.6)		69 (22.8)	
Naturalized	3007 (22.4)	282 (9.4)		578 (19.2)	
Etc.	160 (1.2)	12 (7.5)		37 (23.1)	
**Korean language ability**			0.927		0.001
Fluent	11,069 (82.3)	950 (8.6)		2205 (19.9)	
Fair	2116 (15.7)	177 (8.4)		496 (23.4)	
Poor	258 (1.9)	23 (8.9)		61 (23.6)	
**Employment type**			< 0.001		< 0.001
Permanent	6357 (47.3)	363 (5.7)		1168 (18.4)	
Temporary	3245 (24.1)	309 (9.5)		715 (22.0)	
Day	3841 (28.6)	478 (12.4)		879 (22.9)	
**Company size**			< 0.001		0.087
<5 employees	3059 (22.8)	407 (13.3)		661 (21.6)	
≧5 & <50 employees	7483 (55.7)	563 (7.5)		1487 (19.9)	
≧50 employees	2901 (21.6)	180 (6.2)		614 (21.2)	
**Working hours per week**			< 0.001		< 0.001
<20	393 (2.9)	100 (25.4)		105 (26.7)	
≧20 & <30	583 (4.3)	113 (19.4)		145 (24.9)	
≧30 & <40	885 (6.6)	125 (14.1)		203 (22.9)	
≧40 & <50	7168 (53.3)	434 (6.1)		1322 (18.4)	
≧50 & <60	2194 (16.3)	143 (6.5)		475 (21.6)	
≧60	2220 (16.5)	235 (10.6)		512 (23.1)	
**Occupation**			< 0.001		0.022
White collar	835 (6.2)	23 (2.8)		187 (22.4)	
Pink collar	2524 (18.8)	289 (11.5)		559 (22.1)	
Blue collar	10,084 (75.0)	838 (8.3)		2016 (20.0)	
**Survey year**			< 0.001		< 0.001
2022	4990 (37.1)	490 (9.8)		897 (18.0)	
2020	4634 (34.5)	361 (7.8)		983 (21.2)	
2018	3819 (28.4)	299 (7.8)		882 (23.1)	

^†^P-value of the chi-square test comparing the prevalence of poor self-rated health across different groups

^‡^P-value of the chi-square test comparing the prevalence of ethnic discrimination across different groups

Female Korean Chinese waged workers (12.8%) reported greater poor self-rated health compared to male workers (5.9%). A higher prevalence of poor self-rated health was observed among older workers and in those with lower education levels, longer stays in Korea, those living in metropolitan areas, and those with lower monthly incomes. Regarding occupational covariates, the highest prevalence of poor self-rated health was observed among day laborers, those in workplaces with < 5 employees, those working < 20 h per week, and those in pink-collar occupations.

A higher prevalence of ethnic discrimination was observed among younger workers and in those with higher education levels, lower monthly incomes, and lower level of Korean language ability. For occupational covariates, the highest prevalence of ethnic discrimination was observed among day laborers, those working < 20 h per week, and those in white-collar occupations.

The association between ethnic discrimination and poor self-rated health is presented in Table 2. Ethnic discrimination was associated with poor self-rated health (OR: 1.82, 95% CIs: 1.57–2.10) after adjusting for all covariates, including occupational factors (Model 2). After stratification according to the asking for fair treatment and situational improvements, the “Experienced, not asked for fair treatment” (OR: 1.85, 95% CIs: 1.58–2.15) and “Experienced, asked for fair treatment, not improved” (OR: 1.60, 95% CIs: 1.02–2.53) groups were found to be statistically associated with poor self-rated health after adjusting for all covariates.

**Table 2:** Association between perceived ethnic discrimination and poor self-rated health among Korean Chinese waged workers in South Korea (*N* = 13,443)

Perceived ethnic discrimination	Distribution	Prevalence	Total					
			Unadjusted		Model 1^a^		Model 2^b^	
	N (%)	N (%)	OR	95% CI	OR	95% CI	OR	95% CI
Not experienced	10,681 (79.5)	814 (7.6)	1.00	Referent	1.00	Referent	1.00	Referent
Experienced	2762 (20.5)	336 (12.2)	1.68***	1.47–1.92	1.88***	1.62–2.17	1.82***	1.57–2.10
**Stratified by asking for fair treatment and situational improvements**								
Not experienced	10,681 (79.5)	814 (7.6)	1.00	Referent	1.00	Referent	1.00	Referent
Experienced, not asked for fair treatment	2337 (17.4)	292 (12.5)	1.73***	1.50–1.99	1.91***	1.64–2.22	1.85***	1.58–2.15
Experienced, asked for fair treatment, not improved	264 (2.0)	27 (10.2)	1.38	0.92–2.07	1.67*	1.07–2.62	1.60*	1.02–2.53
Experienced, asked for fair treatment, improved	161 (1.2)	17 (10.6)	1.43	0.86–2.38	1.73	0.99–3.04	1.73	0.99–3.04

*OR* Odds ratio, *CI* Confidence interval

*p < 0.05; **p < 0.01; ***p < 0.001

^a^Adjusted for gender, age, education, length of stay in Korea, residential area, monthly income, marital status, visa type, Korean language ability, and survey year

^b^In addition to Model 1, adjusted for employment type, company size, working hours per week, and occupation

Furthermore, we examined the association between ethnic discrimination and poor self-rated health after stratification by gender (Table 3). Among male workers, the “Experienced, not asked for fair treatment” (OR: 1.85, 95% CIs: 1.47–2.31) and “Experienced, asked for fair treatment, not improved” (OR: 1.86, 95% CIs: 1.04–3.34) groups were statistically significantly associated with poor self-rated health after adjusting for all covariates. On the other hand, among female workers, the “Experienced, not asked for fair treatment” (OR: 1.92, 95% CIs: 1.55–2.37) and “Experienced, asked for fair treatment, improved” (OR: 2.63, 95% CIs: 1.29–5.38) groups were statistically significantly associated with poor self-rated health after adjusting for all covariates.

**Table 3:** Association between perceived ethnic discrimination and poor self-rated health among Korean Chinese waged workers in South Korea by gender (*N* = 13,443)

Perceived ethnic discrimination	Distribution	Prevalence	Unadjusted		Model 1^a^		Model 2^b^	
	N (%)	N (%)	OR	95% CI	OR	95% CI	OR	95% CI
**Male (** ***N*** ** = 8,316)**								
Not experienced	6619 (79.6)	346 (5.2)	1.00	Referent	1.00	Referent	1.00	Referent
Experienced	1697 (20.4)	147 (8.7)	1.72***	1.41–2.10	1.85***	1.49–2.28	1.79***	1.45–2.22
**Stratified by asking for fair treatment and situational improvements**								
Not experienced	6619 (79.6)	346 (5.2)	1.00	Referent	1.00	Referent	1.00	Referent
Experienced, not asked for fair treatment	1433 (17.2)	128 (8.9)	1.78***	1.44–2.20	1.90***	1.52–2.38	1.85***	1.47–2.31
Experienced, asked for fair treatment, not improved	172 (2.1)	15 (8.7)	1.73*	1.01–2.97	1.91*	1.07–3.39	1.86*	1.04–3.34
Experienced, asked for fair treatment, improved	92 (1.1)	4 (4.3)	0.82	0.30–2.26	0.90	0.31–2.60	0.87	0.30–2.53
**Female (** ***N*** ** = 5,127)**								
Not experienced	4062 (79.2)	468 (11.5)	1.00	Referent	1.00	Referent	1.00	Referent
Experienced	1065 (20.8)	189 (17.7)	1.66***	1.38–1.99	1.95***	1.59–2.38	1.90***	1.55–2.33
**Stratified by asking for fair treatment and situational improvements**								
Not experienced	4062 (79.2)	468 (11.5)	1.00	Referent	1.00	Referent	1.00	Referent
Experienced, not asked for fair treatment	904 (17.6)	164 (18.1)	1.70***	1.40–2.07	1.96***	1.59–2.43	1.92***	1.55–2.37
Experienced, asked for fair treatment, not improved	92 (1.8)	12 (13.0)	1.15	0.62–2.13	1.41	0.71–2.82	1.34	0.66–2.72
Experienced, asked for fair treatment, improved	69 (1.3)	13 (18.8)	1.78	0.97–3.28	2.60**	1.27–5.30	2.63**	1.29–5.38

*OR* Odds ratio, *CI* Confidence interval

*p < 0.05; **p < 0.01; ***p < 0.001

^a^Adjusted for age, education, length of stay in Korea, residential area, monthly income, marital status, visa type, Korean language ability, and survey year

^b^In addition to Model 1, adjusted for employment type, company size, working hours per week, and occupation

## Discussion

Our study found that perceived ethnic discrimination was associated with poor self-rated health among Korean Chinese waged workers in Korea, regardless of gender. These findings align with a growing body of evidence highlighting an increased risk of health issues in individuals who experience discrimination [2]. A potential biological pathway through which discrimination affects health is its role as a chronic stressor, where prolonged activation of the body’s stress response system in response to discriminatory norms and experiences can have both immediate and long-term physiological consequences [28].

We also found that 84.6% of Korean Chinese workers who experienced ethnic discrimination did not seek fair treatment in response to experiences of discrimination (84.4% for male workers and 84.9% for female workers). We further found an association with poor self-rated health in this group, regardless of gender. Previous literature has suggested that coping mechanisms such as confrontation may moderate the link between perceived discrimination and health [6]. However, individuals from minority groups with limited social resources due to their lower socioeconomic status often struggle to cope effectively with unfair treatment. This can be attributed to concerns about reprisals, communication challenges, limited social resources and negotiation skills, and insufficient institutional support for direct complaints [29].

Building on the literature that investigated the association between asking for fair treatment after experiencing discrimination and poor self-rated health [20], we divided the participants further among those who asked for fair treatment according to whether the discriminatory situation improved. We found a positive effect on health when the discriminatory situation improves after seeking fair treatment among male workers. However, for female workers, a different pattern emerged, as we observed a statistically significant association with poor self-rated health in the group where the situation was improved. There are three potential explanations for these results. First, a prolonged attempts to confront discrimination until it improves can have adverse effects on female workers’ health. Research has shown that active coping, including direct confrontation, may exacerbate conflict and hostile interactions, potentially worsening the distress caused by discrimination [30]. Particularly for women with limited social resources who persist in seeking fair treatment, the findings suggest that they may endure conflicts, leading to adverse health impacts.

Second, given the vulnerable social position that migrant women often find themselves in, improvements in their situation may indicate the severity of the discrimination that they endure. Women often have limited resources and empowerment to employ effective coping mechanisms when compared to men. Even when women actively seek fair treatment, they may encounter various barriers or experience disregard. For female migrant workers, this challenge can be further pronounced due to the intersectionality of gender and race/ethnicity [31]. This dual minority status places them in a socioeconomically vulnerable position. When we stratified the sociodemographic characteristics of the study participants by gender (Supplementary Table 1), it became evident that women were more likely to earn lower wages and be employed in small-scale businesses with fewer than five employees. This underscores the fact that women often find themselves in relatively disadvantaged working conditions in the labor market compared to men, thereby making it difficult for them to raise their voices and have their requests acknowledged. Given these harsh conditions that female migrant workers often confront, any observed changes in the discriminatory situation may imply a high level of severity of discrimination that demands intervention. Consequently, it is possible that the adverse health effects resulting from serious discrimination could surpass the health benefits of any situational improvement.

Third, female workers may face an increased likelihood of enduring recurrent discrimination, primarily due to gender-based occupational segregation. This segregation stems from the persistence of traditional gender roles, which tend to steer female workers towards face-to-face roles, particularly within the service and care sectors. This segregation may be even more pronounced among Korean Chinese workers due to the host country’s attracting policies. The South Korean government has allowed Korean Chinese under both the VEP and Overseas Korean Visa to work in roles related to housekeeping, childcare, caregiving, and welfare facility support since 2007 and 2010, respectively [32, 33]. As evidenced in the finding of an earlier study, approximately 46% of caregivers in South Korea are foreigners [34], and a significant portion of them are presumed to be Korean Chinese workers, given their proficiency in Korean language and their possession of the necessary visas. Considering the interpersonal nature of face-to-face work, even if these workers have experienced improvements in their discriminatory situations, they remain vulnerable to the possibility of encountering further discrimination. This ongoing risk has the potential to offset the health benefits achieved through the improvement in discriminatory conditions.

This research has several limitations. First, it utilized cross-sectional data, which did not entirely rule out the possibility of reverse causation. However, previous literature has suggested longitudinal evidence showing that racial discrimination can lead to poor health outcomes [35]. Second, we were unable to measure the chronicity, recurrence, intensity, and duration of the discrimination experiences, as indicated in the previous literature [2]. Third, there may be unadjusted confounders, such as baseline health conditions, which may be associated with ethnic discrimination and health outcomes. Lastly, while this study incorporates generalized discrimination as an exposure variable, it specifically focused on discriminatory experiences of waged workers. Thereby not fully capturing comprehensive aspects of ethnic discrimination faced by Korean Chinese immigrants in South Korea. Further research needs to address these limitations by encompassing Korean Chinese immigrants, including self-employed individuals and those who are unemployed.

## Conclusions

To our knowledge, this is the first study to explore the association between perceived ethnic discrimination and poor self-rated health among Korean Chinese waged workers in South Korea. This study also found gender differences in the groups stratified by situational improvements after asking for fair treatment. Subsequent research will be necessary to explain the gender differences.

### Electronic supplementary material

Below is the link to the electronic supplementary material.

Supplementary Material 1

## Data Availability

The datasets used in the study are available to the public: MicroData Integrated Service Website (https://mdis.kostat.go.kr/dwnlSvc/ofrSurvSearch.do?curMenuNo=UI_POR_P9240).

## Abbreviations

SILCLF: Survey on Immigrants’ Living Conditions and Labor Force
VEP: Visit and Employment Program

## References

[1] Braveman P, Egerter S, Williams DR (2011). The Social Determinants of Health: coming of Age. Annu Rev Public Health.

[2] Williams DR, Lawrence JA, Davis BA (2019). Racism and health: evidence and needed research. Annu Rev Public Health.

[3] Devakumar D, Selvarajah S, Abubakar I, Kim SS, McKee M, Sabharwal NS (2022). Racism, xenophobia, discrimination, and the determination of health. Lancet.

[4] Paradies Y, Ben J, Denson N, Elias A, Priest N, Pieterse A (2015). Racism as a determinant of health: a systematic review and Meta-analysis. PLoS ONE.

[5] Lewis TT, Cogburn CD, Williams DR (2015). Self-reported experiences of discrimination and health: scientific advances, ongoing controversies, and emerging issues. Annu Rev Clin Psychol.

[6] Pascoe EA, Richman LS (2009). Perceived discrimination and health: a Meta-Analytic Review. Psychol Bull.

[7] Panza GA, Puhl RM, Taylor BA, Zaleski AL, Livingston J, Pescatello LS (2019). Links between discrimination and cardiovascular health among socially stigmatized groups: a systematic review. PLoS ONE.

[8] Asakura T, Gee GC, Nakayama K, Niwa S (2008). Returning to the homeland: Work-Related Ethnic Discrimination and the health of Japanese brazilians in Japan. Am J Public Health.

[9] Tsuda T, Song C. The cuases of diasporic return: a comparative perspective. In: Tsuda T, Song C, editors. Diasporic returns to the ethnic homeland. Palgrave Macmillan; 2019. pp. 17–34.

[10] Tsuda T, Tsuda T (2009). Global inequities and diasporic return. Diasporic homecomings.

[11] Tsuda T (2022). Racism without racial difference? Co-ethnic racism and national hierarchies among Nikkeijin ethnic return migrants in Japan. Ethnic Racial Stud.

[12] Kwon OJ. Constructing Chineseness as other in the evolution of national identity in South Korea. IDENTITIES-GLOBAL STUDIES IN CULTURE AND POWER. 2021;28(4):454– 71.

[13] Song C. Joseonjok and Goryeo Saram ethnic return migrants in South Korea: Hierarchy among co-ethnics and ethnonational identity. In: Tsuda T, Song C, editors. Diasporic returns to the ethnic homeland. Palgrave Macmillan; 2019. pp. 57–77.

[14] Seol D-H, Skrentny JD (2009). Ethnic return migration and hierarchical nationhood:Korean Chinese foreign workers in South Korea. Ethnicities.

[15] Kim M (2015). The modernity discourse of Folk Culture for the Cultural Integration with the ethnic Korean Chinese [Korean]. J Humanit Unification.

[16] Eum Y-c, Kim J, Noh D-w (2021). Hate Speech and Otherization against Chosun-Jok living in South Korea: focusing on Midnight runners and the outlaws [Korean]. J East-West Comp Literature.

[17] Kim HJ (2017). Exploring stereotypes according to the nationality of immigrants living in Korea [Korean]. Media. Gend Cult.

[18] Korea S. Results of survey on immigrants' living conditions and Labour Force [Korean]. Statistic Korea; 2022.

[19] Seol D-h, Moon H-j (2020). Chapter 4. Korean Migration Management Policy, 1987–2020 [Korean]. Korean Chinese in Korea, 1987–2020.

[20] Kim Y, Son I, Wie D, Muntaner C, Kim H, Kim SS. Don’t ask for fair treatment? A gender analysis of ethnic discrimination, response to discrimination, and self-rated health among marriage migrants in South Korea. Volume 15. INTERNATIONAL JOURNAL FOR EQUITY IN HEALTH; 2016. 10.1186/s12939-016-0396-7 PMC4949882 27430432

[21] Kim Y, Son I, Kim S-s (2015). Association between discrimination and self-rated Health among Marriage migrants in South Korea: focusing on region of origin and gender differences [Korean]. Health Social Welf Rev.

[22] Ra CK, Huh J, Finch BK, Cho Y. The impact of perceived discrimination on depressive symptoms and the role of differentiated social support among immigrant populations in South Korea. Volume 18. INTERNATIONAL JOURNAL FOR EQUITY IN HEALTH; 2019. 10.1186/s12939-019-0910-9 PMC6329122 30634987

[23] Song YN, Jang SH. Transnational ties with the home country matters: the moderation effect of the relationship between perceived discrimination and self-reported health among foreign workers in Korea. Volume 34. ANNALS OF OCCUPATIONAL AND ENVIRONMENTAL MEDICINE; 2022. 10.35371/aoem.2022.34.e18 PMC9436799 36093266

[24] Park GR, Son I, Kim SS (2016). Perceived ethnic discrimination and depressive symptoms among Biethnic adolescents in South Korea. J Prev Med Public Health.

[25] Hong J. Effects of ethnic identity on the Relationship between Mental Health and Perceived discrimination among ethnic return migrants: the case of Korean Chinese return-migrated to South Korea. Volume 21. JOURNAL OF IMMIGRANT AND MINORITY HEALTH; 2019. pp. 522–32. 3. 10.1007/s10903-018-0775-9 29956045

[26] Lee CW. Introduction to Korea’s Survey on immigrants’ living conditions and Labour Force. IOM Migration Research & Training Centre; 2017.

[27] Kim NH. Hierarchical ethnic nationhood in the formal membership and beyond: Joseonjok and formal and substantive citizenshio in their ethnic homeland. In: Tsuda T, Song C, editors. Diasporic returns to the ethnic homeland. Palgrave Macmillan; 2019. pp. 79–97.

[28] Selvarajah S, Corona Maioli S, Deivanayagam TA, de Morais Sato P, Devakumar D, Kim SS (2022). Racism, xenophobia, and discrimination: mapping pathways to health outcomes. Lancet.

[29] Kuo WH (1995). Coping with racial discrimination: the case of Asian americans. Ethnic Racial Stud.

[30] Noh S, Beiser M, Kaspar V, Hou F, Rummens J (1999). Perceived racial discrimination, Depression, and coping: a study of southeast Asian refugees in Canada. J Health Soc Behav.

[31] Crenshaw K (2013). Demarginalizing the intersection of race and sex: a black feminist critique of antidiscrimination doctrine, feminist theory and antiracist politics.

[32] Kim Y-S, National Care Experts and Joseonjok Aunties (2022). Eldercare Workers’ resistance in South Korea [Korean]. Korean Int Migr Stud.

[33] Yuhwi Kim, Lee JA. Study on migrant care workers in South Korea [Korean]. Korea Institute for Health and Social Affairs; 2022. p. 0. Contract No.

[34] Kim Y, Lee J, Ahn S, Park K, Jeon J, Son I. A study on Foreign Care workers in South Korea [Korean]. Korea Institute for Health and Social Affairs; 2021.

[35] Cave L, Cooper MN, Zubrick SR, Shepherd CCJ (2020). Racial discrimination and child and adolescent health in longitudinal studies: a systematic review. Soc Sci Med.

